# When Do Caregivers Talk? The Influences of Activity and Time of Day on Caregiver Speech and Child Vocalizations in Two Childcare Environments

**DOI:** 10.1371/journal.pone.0080646

**Published:** 2013-11-18

**Authors:** Melanie Soderstrom, Kelsey Wittebolle

**Affiliations:** Department of Psychology, University of Manitoba, Winnipeg, Manitoba, Canada; University of Leicester, United Kingdom

## Abstract

The importance of the language environment in influencing language outcomes is well known, but few studies have addressed the contextual factors that influence the amount of speech heard and vocalizations produced by a young child under naturalistic conditions. We analyze effects of type of activity engaged in by the child and time of day on quantitative measures of the language environment. We found effects of both activity and time of day. Structured activities generated the highest levels of adult language, but not necessarily the most child vocalizations. Home and daycare environments looked overall very similar on these measures, however there were important differences across the two environments with respect to the specific effects of activity and time of day.

## Introduction

In the last few decades, there has been an increasing recognition of the importance of the early language environment on children's developmental outcomes. Higher quality language environments are associated with better language outcomes, which in turn are associated with better literacy and scholastic outcomes [1], [2], [3]. Numerous properties and determinants of the language environment have been studied, including but not limited to, the effects of family socio-economic status [2], [3], [4], [5], the influence of different family members and structures [6], and the influence of the childcare environment [7], [8], [9]. The current study examines one possible mediator for many of these effects, namely the types of activity engaged in by and with children during their normal daily lives. Using the LENA system [10], we investigate the manner in which language input varies across these typical daily activities and across the child's day. Using a corpus of full-day, naturalistic recordings, the current study examines the influence of these two potential factors on quantitative measures of child vocalization and adult speech both at home and in a daycare setting.

There are strong links between the amount and quality of language a child hears, and their language development [1], [2], [3], [11]. What is less well understood is what drives the variability in linguistic input. While socio-economic factors have also been identified as playing an important role [5], [12], [13], other kinds of individual differences in environment are likely to also be involved, such as the kind of activities and interactions engaged in by the child and caregiver. Several studies have examined how maternal speech varies across activity type.

Hoff [12] found a complex picture of the effect of four activities (mealtime, dressing, toy play and reading) on language input measures. While reading and dressing activities produced much higher overall measures of the ***rate*** of speech than toy play or mealtime, dressing scored significantly lower than the other three measures on a measure of lexical complexity. In a later analysis of the same data set (excluding dressing), the child's own productions were found to vary in richness across settings, with book reading scoring highest, while the quantity of the child's speech did not differ across settings [14]. Similarly, Rondal [15], found that a number of characteristics of child-directed speech differed systematically across activity contexts, with mealtime eliciting much fewer characteristics of child-directed speech, and less speech overall than free-play and storytime. Lexical diversity in this study was highest in storytime, free-play the lowest. Walker and Armstrong [16] also reported significant effects of context on both form and function of language input, but few details are provided in this report regarding the directions of these effects.

Fewer studies have examined the effect of activity on language measures in external childcare settings, particularly for infants and toddlers. Girolametto and colleagues [17] examined the effect of two activities (book reading and play dough play) in a childcare setting and found systematic differences in measures of directive language input, with book reading associated with a more directive style that inhibited the child's own productions. In a more limited study, Bouchard and colleagues [18] examined a formal characterization of *Language-Support Practices by Early Childhood Educators* in a group of daycares with four-year-old children in Montreal, Canada, during snacktime. Few such practices were found during this activity, though comparisons were not made with other activities.

Two general findings can be gleaned from these studies. First, while details of the contexts and findings differ across studies, it is clear that activity plays an important role in determining the linguistic environment. Second, consistent with a large body of literature within the literacy community, reading-related activities score very highly across a broad spectrum of measures of language quality and quantity, and indeed are associated with better language development [19], [20], [21], although see [22]. Do the effects of activity play out similarly across different types of childcare environment? Which activities are most supportive of language exposure, and which least supportive? How are these activities distributed in children's everyday experiences?

Until now, studies such as those described above have been limited to the examination of small samples of speech, of at most 1–2 hours at a time, limiting their ecological validity. The emergence of the LENA system [10] has provided a powerful new tool to explore these questions. The LENA device is a lightweight, highly durable, wire-free and high quality recording device designed to be worn by an infant or young child as they proceed through their regular day. The LENA system also provides a number of automated estimates of linguistic and other language environment measures. Although the reliability often falls below that of human transcribed data, the advantage of the system is in the ability to collect data samples that reflect much more closely a child's real language experience across an entire day [10], [23].

In a large-scale study of children's language experiences [10], the amount of adult language input heard by a child was found to vary systematically over the course of a child's day, with peaks in the early evening and mid-morning, and a drastic reduction in adult language at mid-day. We therefore also include time-of-day as a variable in our analysis.

We consider our two variables of interest, activity and time of day, across two childcare contexts, the home environment and the centre-based care environment. The current study stems from an ongoing study examining differences between the acoustic and linguistic experiences of children across different childcare settings. Non-maternal childcare is a growing phenomenon in North America [24], [25]. It is therefore crucial to understand the ramifications of these cultural shifts, including the effects on the linguistic environment.

Large-scale studies suggest that the quality of the childcare environment may play a crucial role in early language development. Children in high quality childcare outperform children in maternal care, and children in maternal care outperform children in low quality childcare environments [7], [8], [20], [26]. While these studies do not tend to find an *overall* advantage for centre care over maternal care or vice versa, they are limited to fairly broad measures of the environmental quality and child outcome measures.

A few smaller studies have examined more detailed characteristics of the language environment in daycares [27]–[29], however these studies did not make direct comparisons with home environments. To date, only one study has examined the characteristics of the language environment in early daycare settings in North America specifically in comparison to that of the home environment [9]. This was a case study involving a single high quality daycare, which was compared with data from a previously published study on the home environment [5]. Murray et al [9] found that the quantity of language (e.g. number of words spoken) was comparable to a middle-SES home environment, while language quality (i.e. percentages of declaratives, prohibitions, questions) was comparable to a high-SES home environment. Another study compared a sample of 66 children in France from three groups – maternal care, nanny care, and childcare centre [30]. This study found an advantage for the childcare centre environment over nanny care and maternal care with respect to the quality and quantity of input, although the differences were small and the advantage for centres was not evident across all measures.

Through the course of our data collection in this larger study, it has become increasingly clear that the activity contexts in which these measures are taken are vastly different across childcare settings. While gross quantitative measures may look very similar across these settings, underlyingly the linguistic experiences are very different for a young child of a stay-at-home mother and that of a child placed in full-time centre-based care. Our primary focus in this current study is the effect that differences in activity may have on these linguistic experiences. We will first characterize the types of activity in which children are engaged throughout their day in both the home and the daycare setting. We then consider the effects that these activity types and time of day have on quantitative measures of child vocalization and caregiver speech across two childcare settings (home and childcare centre).

Across these analyses, we are motivated by the following questions: How different or similar are the natural language environments in the home and daycare settings? To what extent is the richness of this language environment driven by activity and time of day? Do these factors play different roles in determining language environment characteristics in the home and daycare setting? Which activity types provide the richest and poorest language input?

## Method

### Ethics Statement

Research was supervised and approved by the University of Manitoba Psychology-Sociology Research Ethics Board. Participating parents, teachers and daycare directors provided written informed consent. Informed consent was provided by the parents both for themselves and on behalf of their children.

### Participants

The participants were eleven children between the ages of 12–29 months in Winnipeg, Canada, recording in childcare centres (N = 6), and home (N = 6) environments, with one child contributing data in both settings (see Table 1).

**Table 1:** Participant Age, Sex and Audio Recording Information.

Child ID	Age: Months	Sex	Environment	Recordings: Date (D/M/Y), Day of Week	Recording Length (Hours: Minutes)**
C001a	18	M	Daycare	10/06/2009, Wednesday	7:25
				26/06/2009, Friday	7:40
C001b	29	M	Daycare	10/08/2009, Monday	7:23
				31/08/2009, Monday	6:57
				01/09/2009, Tuesday	7:07
C002	29	F	Daycare	19/06/2009, Friday	7:00
				22/06/2009, Monday	7:26
				25/06/2009, Thursday	7:43
C021	14	F	Daycare	17/06/2010, Thursday	8:06
				23/06/2010, Wednesday	8:37
				24/06/2010, Thursday	8:34
C024	26	F	Daycare	26/08/2010, Thursday	8:10
				02/09/2010, Thursday	7:17
				08/09/2010, Wednesday	8:01
C022*	19	M	Daycare	22/07/2010, Thursday	8:53
				28/07/2010, Wednesday	8:11
				04/08/2010, Wednesday	8:03
C023*	20	M	Home	21/08/2010, Saturday	9:27
				22/08/2010, Sunday	6:33
				04/09/2010, Saturday	7:05
				25/09/2010, Saturday	8:54
C004	12	F	Home	31/07/2009, Friday	13:57
				10/09/2009, Thursday	11:52
				14/09/2009, Monday	11:38
C005	21	F	Home	25/08/2009, Tuesday	10:04
				26/08/2009, Wednesday	8:36
				02/09/2009, Wednesday	10:00
C006	20	M	Home	06/11/2009, Friday	10:17
				09/11/2009, Monday	10:21
C007	22	F	Home	03/09/2009, Thursday	11:40
				08/09/2009, Tuesday	10:57
				11/09/2009, Friday	10:49
C031	29	M	Home	30/05/2011, Monday	8:11
				16/06/2011, Thursday	8:01
				07/07/2011, Thursday	8:03

*Same child.

**Segments under 5 minutes were excluded from the analysis, so the total length of recording differs slightly from total time used in analysis.

In the childcare centre group, there were three boys and three girls with a mean age of 22.5 months (range 14–29). There were four separate daycares recorded in the study – two of the daycares contributed data from two separate rooms, an “infant” room and a “toddler” room. Since these constituted separate sets of teachers and children and separate physical spaces, they were treated as separate participants in the analysis. In the home group there were three boys and three girls, with a mean age of 20.6 months (range 12–29). Daycare in Winnipeg is highly regulated and comparatively well funded, and our site visits as well as data on teacher certification indicates that all the daycares in our study were high quality. On the other hand, the daycares were distributed in various locations in the city and therefore represented a fairly broad spectrum of the population. Similarly, our home participants were from across the socio-economic spectrum based on data collected regarding maternal education and the neighbourhoods in which our participants resided.

Two additional participants were recorded but not included in the sample. One participant in the home group was partially recorded but not included in the final sample, as her parents withdrew from the study partway through. A second participant was excluded from the home sample because her mother misunderstood our instructions and turned off the recording device during naptime. This artificially inflated the dependent measures and created other technical difficulties with the analysis. A second mother (C006) also turned off the recording device for one sample only – this sample was also excluded from analysis. Only recordings greater than 6 hours were included for all participants.

### Apparatus

The LENA device is a small, unobtrusive device that is placed into a pocket of a vest and worn by the target child. The LENA software system provides automated estimates of a broad spectrum of characteristics of the linguistic environment. The primary quantitative measures generated by the system include estimates of the number of adult words, child vocalizations and conversational turns in a given time window. For the sake of brevity, we will only report directly on the first two measures, as conversational turns is largely derivative of the other two measures. Details of the system are described in the LENA technical reports (available at http://www.lenafoundation.org/Research/TechnicalReports.aspx), as well as in recent peer-reviewed publications based on the system [10], [31].

### Recording Collection Procedure

For the childcare observations, childcare programs throughout the city of Winnipeg were contacted based on a comprehensive online listing of registered childcare programs. Criteria for selecting the “target children” who would wear the device were based on factors such as whether English was the primary language in the home, age of the child, and parent interest, as well as the typical length of a child's stay in the daycare. Each target child was recorded approximately three times. Recording would begin immediately upon a child's arrival in the morning. A research assistant observed the environment for the entirety of the recording, and filled out an observation sheet describing the time of day, numbers of adults and children present, activities, and other comments. The research assistant was instructed not to initiate any interactions with the children, nor promote an interaction initiated by the children in the room. In some cases, children would approach the observer. In such instances, observers engaged in minimal interactions until the child lost interest. The device recorded the child's entire day at the centre. During naptime, the device was removed from the vest and placed near the child's head, but remained active. Childcare programs were given compensation of $50 (or for two centres, $100) per day of recording to be used to purchase toys or to fund specific activities for the children.

Potential home recording participants were found in an existing lab database of parents who had expressed interest in being contacted to participate in research studies on language acquisition. Parents were compensated at $20/day of recording. Parents were asked to fill out their own observation sheets. At the end of each day of recording, the device was returned to the lab to upload the recording.

### Analysis

The LENA software suite (version 3.1.6) was used to generate the three basic quantitative estimates from our audio samples: Adult Word Count (AWC) is an estimate of the raw number of words spoken by any adult in the near presence of the child (approximately 6–10 feet) during a given time period. Child Vocalizations (ChildVoc) is an estimate of the number of times a child makes any kind of linguistically relevant vocalization (i.e. speech or babble, but excluding vegetative noises) during a given time period. Conversational Turns (Turns) is a count of the number of times there is an adult response within 5 seconds of a child vocalization (or vice versa). For all three measures, speech that occurred under noisy conditions (e.g. overlapping with another speaker) or was too quiet/distant was excluded from analysis by the LENA system. All measures were output in 5 minute blocks. In other words, LENA generated data indicating the number of adult words heard by the child from 9:00–9:05, from 9:05–9:10, etc. These counts per five minute block constituted our dependent measures.

Our independent measures consisted of three main variables: the activity category of a given time period, whether the time block took place at home or in a daycare, and time-of-day. The last measure was generated automatically from the LENA system output. The LENA clock and the recorded time on the observational notes did not always agree due to an undetected clock drift in the system. Because the start-stop times listed by mothers doing their own observation sheets were somewhat approximate, we chose to use the LENA clock-time as the Time-of-Day measure. Discrepancies ranged from 0 to 22 minutes, with a mean of 9 minutes. Note that these discrepancies affected the Time-of-Day measure, but *not* the relationship between activity blocks and other measures. The remaining measures were generated from observational data collected at the time of recording, supplemented by retrospective coding by volunteer research assistants.

This retrospective coding was done for two reasons. First, while the original childcare centre recordings involved meticulous note-taking on the part of a research assistant observer present at the time of recording, the observation sheets used for these notes were not designed to study activity per se, and therefore did not contain a systematic set of activity categories. Second, for practical considerations, home recordings relied on the mother to take notes. The mothers' notetaking ranged from highly meticulous to highly haphazard.

Therefore, in order to standardize the activity categories both within and across the samples, each of the original recordings underwent retrospective analysis and recoding based on the audio files and the original observation sheets. For the home recordings, each audio file was listened to in its entirety, and each time segment was assigned a category based on the criteria described below. For the childcare centre recordings, a subset of the audio files were examined to ensure reliability.

For the home recordings, each file was listened to by a volunteer research assistant. For reliability purposes, 216 5-minute segments (in 15 minute chunks) from the home recordings, and 204 from the daycare recordings were recoded by a second volunteer. Agreement was 76% for the home recordings, and 79% for the daycare recordings. Note that this is likely an underestimate of the reliability, since the second coders lacked the full context available to primary coders in making their judgments as they were listening to only partial recordings.

These data were combined with LENA outputs of the dependent variables described above, in 5 minute blocks. Any segment shorter than 5 minutes, usually found at the beginning and end of the recording or any time the device was paused (since time segments were based on clock time), were excluded from any analyses. In total there were 3666 5-minute observation blocks. When entering the activity categories (described below), whichever activity took the majority of the time was used to categorize the 5 minute block, with the exception of diaper changes. Diaper changes were often shorter than 3 minutes, and would therefore not have appeared in the analysis. We allowed for the inclusion of this category by labeling any 5 minute block that included a diaper change, regardless of the length of time it took, as a personal care activity block.

#### Activity categories

Activity of the child at each time point was classified into one of the following categories.

*Playtime* was identified as: the child is playing with a toy, spending time playing with other children, or interacting directly with an adult. Playtime was split into three categories: *outside playtime, organized playtime* and *general playtime*. *Outside playtime* occurred in a setting such as a park or daycare playground. *Organized playtime* entailed an activity that the child would have to sit down for, focus on something or engage in an activity as part of a group. Sing-alongs and painting would both be considered organized playtime. *General playtime* was used when the child was playing with various toys on their own initiative and/or running around. It also served as a catch-all category, for instances when a child was not engaged by adults in a particular activity.

*Storytime* was used when a child listened to one or several books for at least two consecutive minutes, either alone or in a group.

*Mealtime* was used when a child was given food while sitting at a table, in a booster chair, or stopped in a stroller. Mealtime was considered to be over when the child finished eating and/or moved from or was removed from, the location where food was being served.

*Naptime* was counted from when the child was put in the crib or on a bed until they were removed or moved from the sleeping location.

*Transition time* was used to describe general time between other activities. More commonly seen in childcare settings, children will have unstructured time as they go from one activity to the other, such as when they go from playtime inside to a walk outside. Another common example is when children are finished eating, but still in the eating area while daycare staff clean up the other children before playtime can be resumed.

*Outside visits* were only found in the home recordings and encompassed any outside trip except for going outside to play on a designated play structure (i.e. play within a childcare centre's outside play space, or for the home recordings, at a local park, was not counted as outside visit). This category was further subdivided into *family* (a relative's house) and *public* (e.g. office or supermarket).

*Travel time* was used when the children were traveling to an outside visit destination, in any mode of transportation. This category was a bookend to any outside visit, as well as any park time that was coded as outside play.

*TV* as an activity category was used only when the program was a child's program and on with the intent of capturing the child's attention. Note that adult-centred television was not included in this category.

The last major category was *personal care*, which included diaper changes/potty training (getting the child to the changing table/washroom, the activity itself and then any hand washing and rejoining of the group or activity), and bathtime (including the relevant activities leading up to, and following, the actual bath), as well as handwashing outside of meals or diaper changes, dressing, and teeth brushing.

*Other* as a category was reserved for any activity that did not belong to any of the above categories and for which there was not enough similarity with other segments to create a separate category. This included, for example, an incident where a child was examined to determine if they were ill. Only 5 blocks were categorized as other.

## Results

### Distribution of Activities Across Childcare Settings

The nature of activities differed both qualitatively as well as quantitatively in our sample. This was evident both in the kinds of category labels that were appropriate to use in each environment, as well as the kinds of activities that would qualify as a particular label. For example, some activities occurred in only one environment, such as bathtime. Other activities appeared in both environments, but took on a very different character in each. Both children in daycare and those at home engaged in mealtime activities, but the character of these activities was qualitatively very different in the two environments. Mealtime in a daycare setting is a highly ritualized, communal activity, with transition and preparation time, and typically occurs at predetermined times throughout the day. Mealtime (especially snacks) at home is a much more dynamic and fluid activity, and may be overlaid with other activities, including television, playtime etc. The final set of activity categories described above constitutes a compromise between respecting the qualitative differences between activities across the childcare environments and the need to create a set of comparison categories for our quantitative analysis.

**Figure 1:**
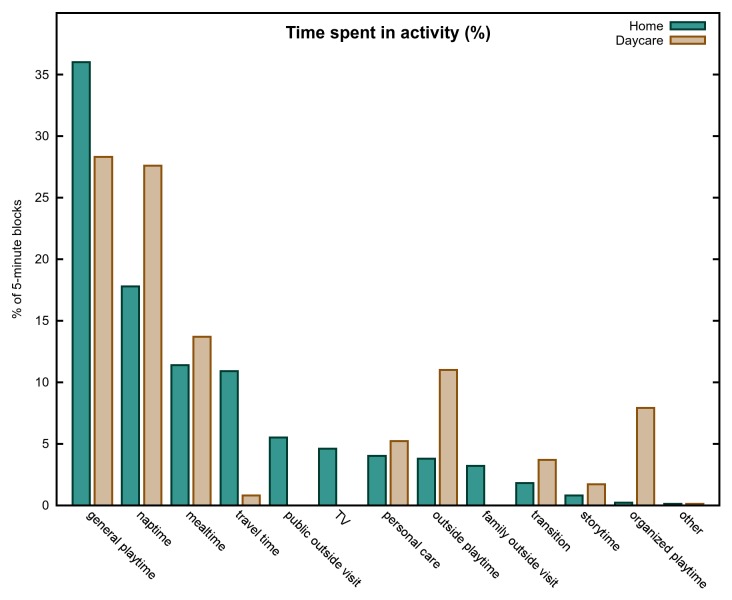
Time spent in activity. The percentage of time spent engaged in each activity type in the two environments.

Figure 1 provides the raw frequency and percentage distributions of the various activities across the daycare and home environments. For both participant groups, general playtime and naptime were the two highest activity categories, constituting together about 50% of the total time in both groups. Mealtime was also a fairly high percentage in both groups, at above 10% of the total time. Storytime was very low in both settings (<2%). Outside playtime (11%) and organized playtime (8%) were also fairly high in the daycare setting, but not in the home setting (5% and 0.2% respectively). These differences between home and daycare were significant by chi-squared comparing the activity in question to all other activities – χ^2^_outside-other_(1, *N* = 2726) = 28.78, *p*<.001, χ^2^_organized-other_(1, *N* = 2736) = 93.14, *p*<.001.

### Effect of Activity and Time of Day Across Environments: General Description of the Analysis

**Figure 2:**
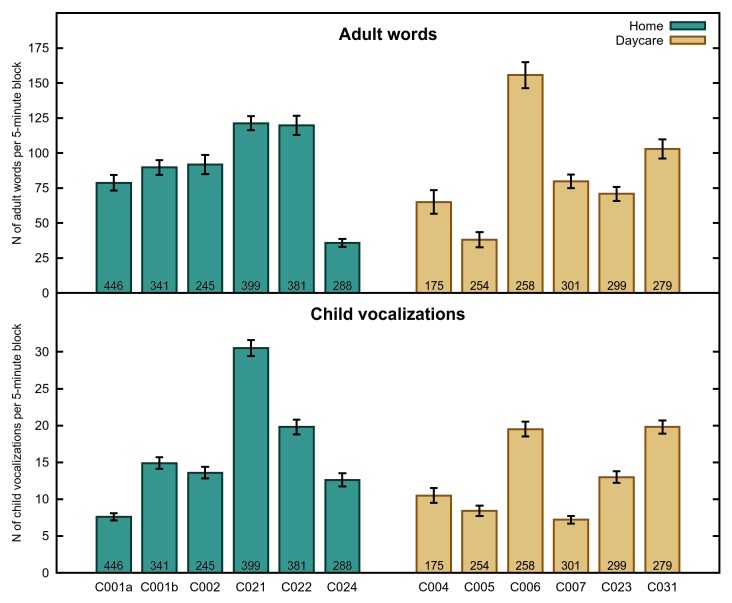
Language Measures by Child. The number of adult words (top) and child vocalizations (bottom) per 5-min block, by child. Error bars indicate standard error. N for each child is indicated at the bottom of their respective bars. C022 and C023 are the same child.

**Figure 3:**
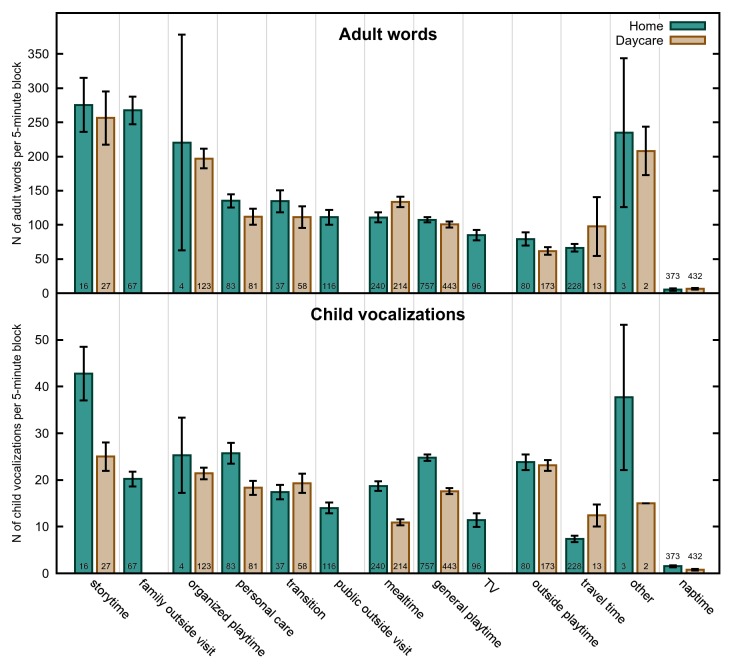
Language Measures by Activity. The number of adult words (top) and child vocalizations (bottom) per 5-min block, by activity. Error bars indicate standard error. N for each child is indicated at the bottom of their respective bars.

**Figure 4:**
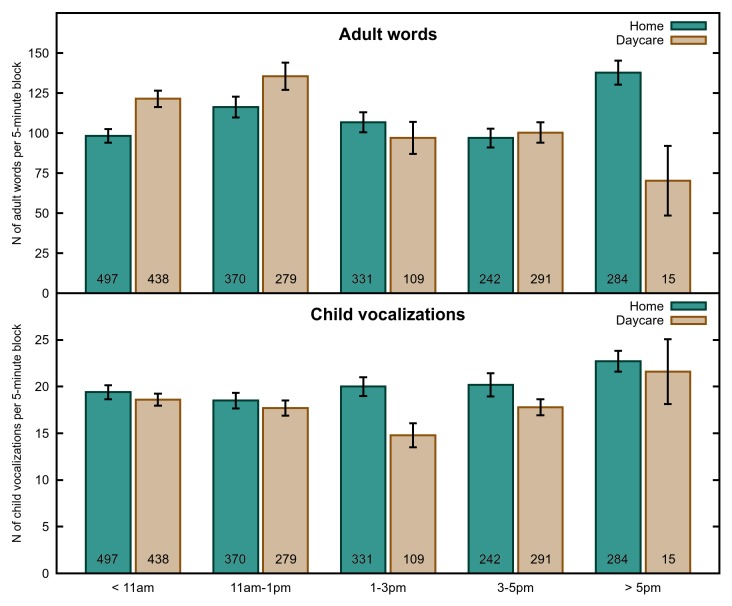
Language Measures by Time of Day. The number of adult words (top) and child vocalizations (bottom) per 5-min block, by time of day. Error bars indicate standard error. N for each child is indicated at the bottom of their respective bars.

We next asked how these differences play out with respect to the language environment of the child. See Figures 2, 3 and 4 for means of our three dependent measures by participant, activity and time of day respectively.

We examined the effect of three categorical variables of interest with respect to each of the dependent measures. They were: environment (2 levels: home, daycare), activity (as described above) and time of day (5 levels defined as follows: before 11 a.m. (AM1), 11 a.m.–1 p.m. (AM2), 1 p.m.–3 p.m. (PM1), 3 p.m.–5 p.m. (PM2) and after 5 p.m. (PM3)).

In determining how to conduct our analysis, we had four special considerations. First, across our various main effects and interactions, there would be a large number of empty cells, due to differences across home and daycare in the activity types engaged in. Second, one activity in particular, the naptime category, was likely to generate highly significant effects of activity for an uninteresting reason – namely that children don't talk and are not spoken to when they are asleep. Third, home recordings were much more likely to start earlier in the morning and run later in the day than daycare recordings, which were largely restricted to the 9 to 5 time frame. This created an imbalance in the time of day measure across our childcare comparison set. Lastly, one participant contributed data in both groups, which means that our dataset was partially within-participants and partially between-participants. For these reasons, we ran most of our analyses on the daycare and home datasets separately, and excluded the naptime variable from all of the statistical analyses reported below (the *other* category was also excluded from analysis). Any analyses including both daycare and home data excluded participant C022/C023's home data.

Our dataset involved two types of random variables (5 minute activity blocks and participant child). To allow for this, we employed a series of linear mixed model analyses, using SAS 9.2, separately for each of our dependent variables (AWC and ChildVoc). The participant child was listed as the “subject” for all analyses. For each dependent measure, our first analysis included all three of our main effects and all interactions. In this analysis we constrained the dataset to recording times between 9 a.m. and 5 p.m., and excluded TV and outside visits (in addition to naptime and other) since these activities were not found in the daycare environment. In this analysis, C023 data (i.e., the within-subject home recording) was excluded to avoid the issue of partial-within data. We then ran separate analyses for the daycare and home environments for the main effects of activity and time of day. These analyses included the C023 data. After each model, we explored these main effects using contrast analyses.

### Adult Word Count (AWC) Analysis

Our first analysis used the restricted dataset as described above. This model included the variables time of day, activity, and environment, with all main effects and interactions included. We found a significant effect of time of day (F(3, 27) = 6.52, p = .0019), a marginal effect of activity (F(7, 46) = 1.92, p = .0872), and no significant effect of environment (F(1, 9) = .82, p = .3886). All four interactions were significant: Time of Day X Environment (F(3, 27) = 3.39, p = .0323), Time of Day X Activity (F(21, 60) = 3.55, p<.0001), Environment X Activity (F(7, 46) = 6.56, p<.0001) and the three-way interaction (F(15, 60) = 3.45, p = .0003).

We then examined the home and daycare environments separately, with the fuller dataset (including all time segments, C023 and excluding only *naptime* and *other* activities). The model design was identical but for the exclusion of the environment main effect and interactions. For the daycare analysis, both main effects and the interaction were significant (time of day: F(4, 16) = 7.75, p = .0011; activity: F(7, 28) = 11.05, p<.0001); Time of Day X Activity interaction: F(23, 44) = 4.72, p<.0001). For the home analysis, the time of day main effect was not significant (F(4, 19) = 1.91, p = .1498). However, activity was significant (F(10, 38) = 11.47, p<.0001) as was the interaction between activity and time of day (F(36, 47) = 2.57, p = .0013).

#### Analysis of Time of Day

Since there was no significant effect of Time of Day at home, we examined only the daycare setting. In this analysis, the AM2 time period (11 a.m. to 1 p.m.) was the highest ranked time of day, significantly higher than the other time periods combined (F(1, 16) = 10.80, p = .0046). This is exactly the opposite of the effect found by Greenwood and colleagues [10], for whom this was the lowest time period. However, it is important to note that when the naptime category is included in the analysis the data shift so that our data look much more similar to that of Greenwood et al. In other words, the sleep patterns of the children play a significant role in determining the effect of time of day on AWC, and may well account for the pattern found in the Greenwood et al. study. Nonetheless, there appear to be time of day effects on the amount of speech children hear even during awake time, at least in some contexts.

#### Analysis of Activity

Despite a significant Environment X Activity interaction in the combined analysis, the daycare-only and home-only analyses looked very similar overall. In both analyses, storytime was the highest-ranked activity, followed by organized playtime. In the daycare setting, these two were significantly different from each other, (F(1, 28) = 14.45, p = .0007), although at home they were not (F(1, 38) = 1.27, p = .2672). The home-only activity family outside visit was also not significantly different from storytime or organized playtime. In the daycare analysis, organized playtime was significantly higher than the next highest ranked, mealtime (F(1, 28) = 15.51, p = .0005), although this was only marginally significant in the home analysis (F(1, 38) = 3.47, p = .0701). Mealtime, personal care, and transition time were all similar in their AWC, in both analyses. Mealtime was significantly higher in AWC than general playtime in the daycare analysis, (F(1, 28) = 8.77, p = .0062), but only marginally so in the home analysis (F(1, 38) = 3.51, p = .0688). In both analyses, travel and outside playtime were the two activities with the lowest AWC and not significantly different from each other, although in the home analysis, this was closely followed by TV. In both analyses, general playtime was significantly higher in AWC than outside playtime (daycare: F(1, 28) = 17.55, p = .0003; home: (F(1, 38) = 5.56, p = .0236).

### Child Vocalization (ChildVoc) Analysis

As above, we used a time and activity-restricted dataset and included the variables time of day, activity, and environment, with all main effects and interactions included. In this analysis, we found a significant effect of activity (F(7, 46) = 11.97, p<.0001), but no effect of environment (F(1, 9) = .95, p = .3557) or time of day (F(3, 27) = 1.06, p = .3837). All two-way interactions were significant, however, (Time of Day X Environment: F(3, 27) = 1.03, p = .3936); Time of Day X Activity: F(21, 60) = 1.84, p = .0337); Activity X Environment: F(7, 46) = 2.99, p = .0113). The three-way interaction was not significant (F(15, 60) = 1.24, p = .2680).

For the daycare analysis, activity was significant (F(7, 28) = 8.93, p<.0001), but the main effect of time of day (F(4, 16) = 2.73, p = .0663) and the interaction (F(23, 44) = 1.65, p = .0756) were both marginal. For the home analysis, both main effects (time of day: (F(4, 19) = 2.93, p = .0480; activity: (10, 38) = 16.27, p<.0001) and the interaction (F(36, 47) = 1.75, p = .0363) were significant.

#### Analysis of Time of Day

For the daycare analysis, PM3, PM2, AM2 and AM1 had significantly more child vocalizations than PM1 (F(1, 16) = 13.86, p = .0018) but not from each other. For the home analysis, the highest ranked time (PM3) was significantly higher in child vocalizations than the other four times combined (F(1, 19) = 8.00, p = .0107), but did not significantly outstrip the next highest time period (PM2) in a direct comparison (F(1, 19) = .52, p = .4794). Similarly, the lowest ranked time (AM2) was significantly lower than the other time periods combined (F(1, 19) = 9.06, p = .0072), but not than AM1, the next-lowest time period (F(1, 19) = .21, p = .6552.

#### Analysis of Activity

The home and daycare analyses of ChildVoc by activity showed some more notable differences. While in both analyses storytime was the highest-ranked activity, the number of child vocalizations in the home environment was almost twice as much as in the daycare environment. In the daycare analysis storytime was not significantly different from the next-ranked activity, outside playtime (F(1, 28) = .01, p = .9266), and was only marginally higher than organized playtime (F(1, 28) = 3.26, p = .0819), versus in the home analysis in which storytime generated significantly more vocalizations than the next-ranked activity, personal care (F(1, 38) = 11.25, p = .0018 – although see below regarding a possible reliability issue in this finding).

Similarly, while travel time was ranked very low in both environments, mealtime was ranked lowest for the daycare analysis only, significantly lower than general playtime (F(1, 28) = 58.92, p<.0001). In the home analysis, mealtime was mid-ranked (with again almost twice as much vocalization as in the daycare environment). In the home environment, as noted above, personal care was ranked second-highest after storytime, while in the daycare environment, this was a mid-range activity. In fact, personal care in the home environment was numerically higher in child vocalizations than the highest-ranked activity at daycare (i.e. storytime).

One activity that is striking different from the AWC analysis is that of outside playtime, which ranked very highly in ChildVoc in both the daycare and the home analysis.

### Human-coding comparison with LENA measures

Although there are existing measures of reliability for LENA, as discussed above, it is known to be less reliable in certain contexts, such as noisy conditions. This would be problematic for our findings if the LENA system's reliability is reduced in ways that skew the data differently across the contexts we are studying. We therefore hand-coded 183 of the 5-minute blocks, and compared these hand-coded measures of adult word count and number of child vocalizations to the LENA-generated numbers. We limited the selection to segments from 9 a.m. to 5 p.m. in order to better compare the reliability across chilcare settings. Within this timeframe, there were only 3 blocks of organized playtime in the home.

The hand-coder was a research assistant in the lab (not one of the authors) who was familiar with the general goal of the project but not with the specific details of the outcome with respect to particular activities, nor with where specific differences and similarities were found between environments. The coder was aware of whether a specific block being coded was from a daycare or a home, but not given direct information about what activity category this block had been assigned during the analysis. The coder was also blind during the coding process to the specific values for Adult Word Count and Child Vocalizations assigned by LENA. A computer script randomly selected the 20 blocks for each category based on the data spreadsheet, and made the 5-minute audio block available to the coder. The hand-coder then listened to the audio file and coded each 5-minute block in two separate passes. In the first pass, the coder transcribed all of the adult words heard during the 5-minute block. Adult words heard during significant noise (including overlapping speech) were excluded to be consistent with LENA parameters for the adult word count. The script then converted this transcription into a word count based on white space between words. In the second pass, the coder counted the number of child vocalizations. A child vocalization was any speech-like or babbling vocalization. Multiple vocalizations were treated separately if there was significant pausing in between (approximately 1 s). Vegetative noises, laughter and crying were not counted as vocalizations.

These data are reported in Table 2. We also calculated a Pearson coefficient between the LENA and human-coded data. For the adult word count, there was an overall correlation of r = .764, N = 183, p<.001, a correlation of r = .769, N = 100, p<.001 for the daycare data, and r = .828, N = 83, p<.001 for the home data. For the child vocalizations the overall correlation was r = .688, N = 183, p<.001, for daycare data r = .716, N = 100, p<.001, for home data r = .646, N = 83, p<.001. Another way of looking at the reliability of the data is to compare the means/rank order of the hand-coded and LENA-based data. For adult word count, the picture is very similar regardless of which analysis is used for both daycare and home environments. In the daycare data subset, organized playtime is by far the highest measure, mealtime the next highest, and outside playtime the lowest, general playtime and personal care in between. For the home data subset, personal care was the highest measure, followed by outside playtime, with the other three measures lower. Note that these relative differences do not match exactly what was found in the larger dataset. What is important is that the two ways of measuring the data (LENA and hand-coding) appear to generate very similar findings. This also appears to be true of the daycare child vocalization measures. Organized playtime and outside playtime generated much larger counts of child vocalizations for both measures than the other three activities. We did find some discrepancies in the home data, however, reflecting the lower reliability measured above. Organized playtime and personal care both generated higher rates of child vocalization in the hand-coded data than the LENA data. However, organized playtime was based on an extremely small sample from only two participants, limiting its interpretability outside of the larger analysis. It is also worth noting that both of these activities ranked high in the original sample. It is therefore possible that the LENA analysis underestimates the amount of child vocalization in these activities at home, but the overall finding that they generate a high number of child vocalizations is not challenged by the reliability discrepancy. Nor do these data support the idea that child vocalizations are radically overestimated by LENA in the daycare setting (although some degree of overestimation may be occurring), despite the presence of other same-aged children. In sum, although the reliability between LENA measures and hand-coding was somewhat modest, there was consistency across these two measures, and little evidence that failures in the LENA system generated substantive artifacts in our findings.

**Table 2:** Comparison of LENA and Human Coder measures of Adult Words and Child Vocalizations.

Daycare Environment	LENA	Transcriber	LENA	Transcriber
	Adult Words	Adult Words	Child Vocaliations	Child Vocalizations
Mealtime	125.2	97.4	11.5	13.4
Personal Care	80.4	68.8	13.8	16.2
Playtime – General	84.7	50.4	13.4	18.6
Playtime – Organized	275.6	164.9	24.9	24.6
Playtime – Outside	30.4	41.6	24.9	25.2

Home Environment	LENA	Transcriber	LENA	Transcriber
	Adult Words	Adult Words	Child Vocaliations	Child Vocalizations
Mealtime	90.7	65.7	21.8	20.9
Personal Care	137.9	155.8	21.7	28.2
Playtime – General	97.4	66.0	28.1	28.0
Playtime – Organized	86.0	67.8	20.0	32.3
Playtime – Outside	107.8	119.3	19.3	18.6

N = 20 for all categories except organized playtime at home (N = 3).

## Discussion

This study provides a window into some of the factors influencing the quantity of linguistic input to young children both in the home and at daycare. Across all analyses, the activity in which a child was engaged showed the most robust influence on the richness of that input, while the time of day overall had less robust influences, particularly at home. There was no evidence of an overall main effect of Environment in our data. This is consistent with large scale studies show no overall effect of amount or type of childcare on language development [7], [8], [32].

### Time of Day Effects on the Language Environment

Consistent with Greenwood et al. [10], we found significant effects of time of day on the quantity of language a child hears, however the mid-day effects reported in the Greenwood study seem to be largely driven by children's nap patterns. When naptime is removed from the analysis, there remain some influences of time of day, however these vary across childcare settings. It is important to note that our datasets did not include very many data points during the evening hours, which was the point of highest adult language in the Greenwood study.

Time of day effects on the linguistic environment are different from some other potential effects, in that if they are truly direct effects (e.g., due to biological rhythms in arousal) they are relatively immune from manipulation. However, another possibility is that they are a stand-in for other effects, such as the usual timing of certain kinds of activities. The naptime effect is a good example of this latter possibility. While our data do not allow us to tease apart these possibilities directly, the strong role that activity type plays in determining linguistic input suggests that this indirect effect is at least partially at play. In other words, the limited time of day effects that we did find once naptime was excluded may well have been the result of particular activities engaged in at scheduled times of day – particularly in the daycare setting in which activities tended to be highly structured. At the very least, our findings suggest that researchers need to be cautious about the possibility of time of day effects (as well as activity) in comparing across samples recorded at different times.

### Effects of Activity on the Language Environment

Consistently across our analysis, storytime and organized playtime were the highest, or among the highest, activities with respect to quantitative measures of language input. Our data therefore support the existing literature suggesting that structured activities, and in particular, reading-based activities, provide a boost to the quantity of language heard by a child. Our findings go beyond this literature in some important ways. For one, while the AWC was similar across child care settings, ChildVoc during storytime was about twice as high at home than at daycare. Although these differences are based on a relatively small N in both settings, they are striking, and suggest that the storytime activity may be of a different, more interactive, character at home than in daycare; see also [17]. Likely this has to do with storytime being a one-on-one activity at home, while it is typically a large-group activity in daycare. Relatedly, work in [33] provides a comprehensive analysis of the character of book-reading and other language-supporting activities at home and preschool in slightly older children. It is noteworthy that while storytime was by far the most productive with respective to our quantitative measures, it was one of the smallest measures with respect to frequency, constituting only 1–2% of a child's daytime experience in our sample. This begs the question of the extent to which daytime reading activities typically influence language development, if they are so rare. It is possible that small amounts of storytime have strong effects on linguistic development despite their relative rarity, particularly in households that do not have bedtime reading rituals (note that most of our recordings did not include bedtime activities). However, it is also possible that other, more common activities with high or moderate linguistic output and social interaction, such as personal care and playtime, may be more crucial activities in determining the linguistic environment during the day.

Our study also allows us to examine the mealtime activity. While the studies described in the introduction generally find it to be one of relatively low language input, others [33] have argued that it provides a unique opportunity for language interactions. Our analysis of mealtime is consistent with the previous research in that it is not among the highest-ranked activities. It was generally a middle-ranked activity, with the exception of the child vocalization in the daycare setting, in which it was ranked quite low. Age may well play a role here, as older children may be more likely to be engaged in narrative interactions at mealtime, while infants and toddlers may be more focused on the task of learning how to eat.

Another activity that differed across child care settings was that of personal care. While this activity generated relatively average levels of adult language for both groups, the quantity of child vocalizations was quite high in the home setting, but not for the daycare setting. This suggests that personal care activities result in more interactive language in the home setting than at daycare.

Another activity that scored very high for the AWC measure in the home environment (this activity was obviously not found in the daycare recordings) was family outside visit – i.e. visits to others' homes. Interestingly, this activity was somewhat lower in terms of child vocalizations, suggesting that visits to others' homes generated a high level of talk between adults, but not necessarily a correspondingly high level of child vocalization, child-directed speech or child engagement.

Looking at the other end of the scale, some of the lowest counts for both groups were travel time. Another activity with fairly depressed numbers was outside playtime. This activity generated fairly low levels of AWC, but much higher measures of ChildVoc. It is important to keep in mind with respect to this measure that outdoor recordings are likely to be somewhat poorer quality (given ambient wind noise, different acoustics, etc.) than indoor recordings, so it is possible that one factor in the depressed numbers for this activity is simply less capability of the LENA system to adequately capture or identify meaningful language. However, it is likely that this result is also capturing a true effect of fewer linguistic interactions with adults when children are playing outside, particularly given that the ChildVoc measure was comparatively stable.

Our findings may provide insight into to the types of activities that best support language development. The most obvious of these is the advantage of structured playtime (including book reading) over unstructured playtime in quantity of linguistic input. However, we hesitate to suggest this as directly informative of best practices, particularly in child care contexts. While this study provides a *quantitative* measure of linguistic input, we do not know the degree to which these differences in quantity translate (or fail to translate) into outcomes with respect to language development. Furthermore, given the growing evidence that interaction plays a crucial role in language acquisition [34], [35], it is worth noting that child vocalizations were relatively depressed for this activity in the daycare environment compared with at home. Notably, Yont et al. [22] similarly found reduced child language during book reading compared with free play in their analysis of semi-structured mother-child interactions in the home. Nor have we yet examined any direct *qualitative* measures of language input, such as proportion of child-directed speech. Therefore, while increasing the amount of structured play and storytime in daycares might increase the raw level of adult language input, this might not result in a qualitatively better linguistic environment. This illustrates an important implication of this study, namely that we must be cautious about interpreting findings at home as being relevant to the daycare environment and vice versa.

### Limitations of the Study

While this study provides important insights into the nature of the relationship between various factors of the child's environment and the linguistic input, there are some crucial limitations which must be considered. First, the study was relatively small in scope due to the effort involved in collecting our independent measures (although it was much larger than the only comparative study of its kind, Murray et al. [9]), and the sample was fairly constrained with respect to both the type and quality of the daycares and the participant group. Therefore, our findings may be restricted to the set of relatively high quality daycares such as those found in Winnipeg, Canada, and the relatively homogeneous group (at least with respect to ethnicity) that was our home comparison sample, particularly given the well-known relationship between SES and language input [2]–[5].

Second, there are important technical limitations of our measures. Our dependent measures relied entirely on the estimates provided by the LENA system and are therefore vulnerable to any errors or weaknesses in this system. The LENA technical reports and recent published findings using LENA cited above, plus our in-house reports provide good reason to believe that these estimates are a reliable representation of the language input to children. One important source of error relevant to the current study is the known reduction in reliability under noisy conditions. Since daycares are known to be relatively noisy compared with home environments, there may be a systematic difference in reliability across the two samples (as well as across different activities with different noise levels). However, this reduction in reliability would primarily be reflected in a reduction of the quantitative values of our dependent measures under noisy conditions. This is in fact just what one would want to reflect a decrease in likelihood that a particular utterance would be heard by a child under noisy conditions, particularly given that infants have reduced perceptual capabilities under noisy conditions compared with adults [36]. On the other hand, the extent to which infants process speech under such conditions of noise, including overlapping speech, is still an open question, so LENA may significantly underestimate the amount of speech heard under these conditions, which could skew our findings.

Finally, as discussed above, it is important to keep in mind that this study analyzed only quantitative measures of linguistic input, and not qualitative measures. These weaknesses are offset by the larger, more naturalistic, set of data we were able to analyze due to the automated dependent measures. They constitute the first in what we hope to be a growing literature using LENA to provide large-scale, naturalistic data convergent with more traditional laboratory and hand-coded studies.

## Conclusions

This study examined the effect of activity type and time of day across two childcare settings (home and daycare centre) on the amount of caregiver and child vocalization. Activity, and to a lesser extent time of day, had significant effects on the quantity of linguistic input in a child's daytime experience both at home and in daycare. While we did not find evidence that the childcare environment influenced the linguistic input measures, there were important interactions between environment and some of the other factors, suggesting that there may be qualitative differences between the home and daycare environment in the linguistic experiences of the child.

The study raises a number of important questions for future consideration. For example, what are the crucial factors that underlie the differences between the home and daycare activities and across the activities? We have identified a few possibilities, but this is open to empirical investigation and there are other possibilities. To what extent do these differences in quantity reflect factors that determine child language outcomes? Do the measures of caregiver and child vocalizations differ in their importance for language development?

These finding constitute the first systematic attempt to analyze these factors together in a single study under naturalistic conditions, and demonstrate that time of day and activity have important influences on the linguistic environment. In addition, while childcare environment did not significantly influence these measures (at least in this small sample), there were important differences found in the manner and extent to which these other factors influenced the language environment.
